# Primary care-based interventions to address the financial needs of patients experiencing poverty: a scoping review of the literature

**DOI:** 10.1186/s12939-021-01546-8

**Published:** 2021-10-07

**Authors:** Jane Parry, Meredith Vanstone, Michel Grignon, James R. Dunn

**Affiliations:** 1 Department of Health, Aging and Society, McMaster University, 1280 Main St West, Hamilton, ON L8S 4L8 Canada; 2 Department of Family Medicine, McMaster University, 1280 Main St West, Hamilton, ON L8S 4L8 Canada

**Keywords:** Primary care, Poverty, Patients, Health equity, High-income country, Health care providers, Health care delivery, Scoping review

## Abstract

**Background:**

It is broadly accepted that poverty is associated with poor health, and the health impact of poverty has been explored in numerous high-income country settings. There is a large and growing body of evidence of the role that primary care practitioners can play in identifying poverty as a health determinant, and in interventions to address it.

**Purpose of study:**

This study maps the published peer-reviewed and grey literature on primary care setting interventions to address poverty in high-income countries in order to identify key concepts and gaps in the research. This scoping review seeks to map the tools in use to identify and address patients’ economic needs; describe the key types of primary care-based interventions; and examine barriers and facilitators to successful implementation.

**Methods:**

Using a scoping review methodology, we searched five databases, the grey literature and the reference lists of relevant studies to identify studies on interventions to address the economic needs-related social determinants of health that occur in primary health care delivery settings, in high-income countries. Findings were synthesized narratively, and examined using thematic analysis, according to iteratively identified themes.

**Results:**

Two hundred and fourteen papers were included in the review and fell into two broad categories of description and evaluation: screening tools, and economic needs-specific interventions. Primary care-based interventions that aim to address patients’ financial needs operate at all levels, from passive sociodemographic data collection upon patient registration, through referral to external services, to direct intervention in addressing patients’ income needs.

**Conclusion:**

Tools and processes to identify and address patients’ economic social needs range from those tailored to individual health practices, or addressing one specific dimension of need, to wide-ranging protocols. Primary care-based interventions to address income needs operate at all levels, from passive sociodemographic data collection, through referral to external services, to direct intervention. Measuring success has proven challenging. The decision to undertake this work requires courage on the part of health care providers because it can be difficult, time-consuming and complex. However, it is often appreciated by patients, even when the scope of action available to health care providers is quite narrow.

## Background

The social determinants of health are defined by the World Health Organization as the “conditions in which people are born, grow, work, live and age, and the wider set of forces and systems shaping the conditions of daily life” [1]. It is broadly accepted that poverty is associated with poor health [2]. The influence of money on health can be examined according to multiple theories, including material mechanisms, psychosocial and behavioural pathways, and the impact of disability on income, and can be conceptualized as a combination of more than one pathway [3–9]. The health impact of poverty has been explored in numerous high-income country settings. In Canada, for example, while social policies, notably universal health insurance, attenuate the negative relationship between low income and health, those in the lowest income quintile have higher rates of chronic disease and disability, and there is some evidence that income interventions may improve health at a population level [2, 10–14]. However, whether or not health care providers and the health care system can–or even should–play a part in addressing them remains contested [15–17].

Nested within the larger societal conversation about the social determinants of individual and population health, there is an ongoing discussion happening in both the public and academic arenas about the role of primary care in addressing them [18–21]. Primary care, i.e., the services of a doctor that patients can access directly, without referral, and which are not offered in an emergency setting, is typically the first point of contact in the health care system, aiming to provide continuous, comprehensive, and coordinated care [22]. Primary care is delivered in different health care settings, and can even be in non-health sector settings, such as schools [23, 24]. The primary care concept, according to WHO, is “a whole-of-society approach to health and well-being centred on the needs and preferences of individuals, families and communities. It addresses the broader determinants of health and focuses on the comprehensive and interrelated aspects of physical, mental and social health” [25]. As such, there is a strong argument that poverty, as a SDOH, is well within the remit of primary care. Indeed, in the health care sector, primary care in particular has been a setting for interventions to address poverty [26–30].

Primary care involvement in addressing SDOH varies in both depth and scope. In terms of depth, it can be in the form of screening, with or without subsequent referral to services to address identified needs. It can extend to interventions within the primary care setting itself, beyond signposting for external supports. Social prescribing is one commonly used term for such interventions, but there is no universally accepted definition of this term, or consensus on what it encompasses [31]. While this study includes articles on social prescribing, it specifically examines interventions that aim to directly improve the client’s economic circumstances. In terms of scope, such interventions can focus on a particular SDOH domain, such as housing [32], income [33], or education [34], or it can be broad-ranging, covering multiple SDOH and even incorporating behavioural and psycho-social aspects of individual health [35].

There are sceptics on the role of health care providers in addressing the economic needs of patients living in poverty, including those who argue that social justice is beyond the scope of medical practice [36]. There are also concerns that those who are most in need of healthcare services are the least likely to have access to them [37]. As such, interventions to address poverty could widen inequities, not narrow them [38]. Another concern is that in the process of screening for social needs, health care providers will be faced with problems that they do not have the resources to address, or will create unfulfilled expectations for patients, and may also take up time that could otherwise be spent on clinical care [39, 40]. Addressing economic needs in primary care may also distract from inadequacies in the social safety net that bring those needs into the doctor’s clinic in the first place [41]. There is also evidence to suggest that even if patients disclose non-medical needs to their primary care provider, they may not want clinicians’ help to address those needs [42].

These criticisms notwithstanding, there is a large and growing body of evidence to demonstrate that individual medical practitioners encounter the embodiment of poverty in their patients, and see addressing patients’ socio-economic as part of their remit as health care providers, and that some health care organizations are choosing to address them [32, 43–47]. While there is a plethora of literature on various aspects of primary care-based interventions to address poverty, what is missing is an understanding of the over-arching themes that can be gleaned from this vast body of literature, such as the scope, target users and format of screening tools, and the types of interventions and what they specifically aim to address. Investigating this can also highlight areas where the field would benefit from more research.

### Objectives

The purpose of this study is to map the published peer-reviewed and grey literature on primary care setting interventions to address poverty in high-income countries in order to identify key concepts and gaps in the research. There are many different ways to screen for and intervene in patients’ economic needs, and the inclusion criteria were deliberately constructed to capture the heterogeneity of such screening tools and interventions. Unlike previous studies, this study focuses specifically on interventions targeting economic needs, and investigates interventions in the primary care setting across the whole spectrum, from screening patients, collecting and managing the data generated in the process; referring patients to external services, and directly intervening to address patients’ needs. In the process of examining the literature, this scoping review seeks to map the tools in use to identify and address patients’ economic needs; describe the key types of primary care-based interventions; and examine barriers and facilitators to successful implementation. Its breadth of scope differentiates it from previous systematic reviews and scoping reviews, which have looked specifically at, for example, the impact of social needs interventions on health outcomes and spending [21], screening tools [48–50], social prescribing and system navigation [51–53], or which have examined SDOH more broadly [54]. This review will be global in scope, rather than concentrated on the US, as is the case in other studies [55].

## Methods

The scoping review study design was selected because it is the one that is well suited to a topic where the literature is vast, complex, and heterogeneous, including theoretical and narrative reviews, and quantitative, qualitative and mixed-methods studies, peer-reviewed and grey literature [56, 57]. The aim is to map key concepts and clarify working definitions rather than to address a precise question, such as measurable outcomes from a particular type of intervention [58]. Scoping reviews are useful for revealing the ‘lay of the land’ [59]. There is no universally accepted definition of what constitutes a scoping review; although there are no highly rigid structures for conducting one, a scoping review must still be systematic, reproducible and accountable [60].

This scoping review uses the six-step Arksey and O’Malley framework for conducting scoping reviews: identify the research question; identify the relevant studies; select the studies for review; chart the data; then collate, summarize and report the findings. There is an optional step to make recommendations [61]. It follows Tricco et al’s Preferred Reporting Items for Systematic Reviews and Meta-Analyses extension for Scoping Reviews checklist [62].

### Search strategy

#### Selecting the literature

For scoping reviews, the challenge is how to strike a balance between the breadth and comprehensiveness of the available literature, vs. the resources available to conduct the study [57]. This is overcome by placing limitations around the scope of the searches, guided by the research questions and an initial review of the literature, in an iterative fashion [59]. For this scoping review, search inclusion criteria were English-language published peer-reviewed and grey literature published from January 12,000 to the date of the search (between August 7 and 212,020, with the last search conducted on 21 August 2020.) For inclusion, a screening, referral or intervention paper had to include at least one of the following terms in the title or abstract: SDOH, income, employment, food security/insecurity, housing/homelessness, legal services, education, transport and be related to the clinical health care services delivery system. Programmes had to be delivered within the primary care clinical setting, either by a health care professional, dedicated staff member or volunteer. The inclusion criteria thus targeted the search to health care setting interventions, rather than community-level interventions, or those in other settings such as welfare rights centres or schools. Studies were excluded if they did not meet these criteria, and if they were not related to economic security needs screening, referral or intervention. By searching the literature using the key words ‘primary care’, ‘family practice’ and/or ‘health centre/center’, it was possible to include primary care settings, which in one context would count as primary care (e.g. pediatricians in the US), that would not in another, and include settings such as community health centres, (which exist in Canada and the US, but which do not exist in the same form in the UK, for example).

**Fig. 1:**
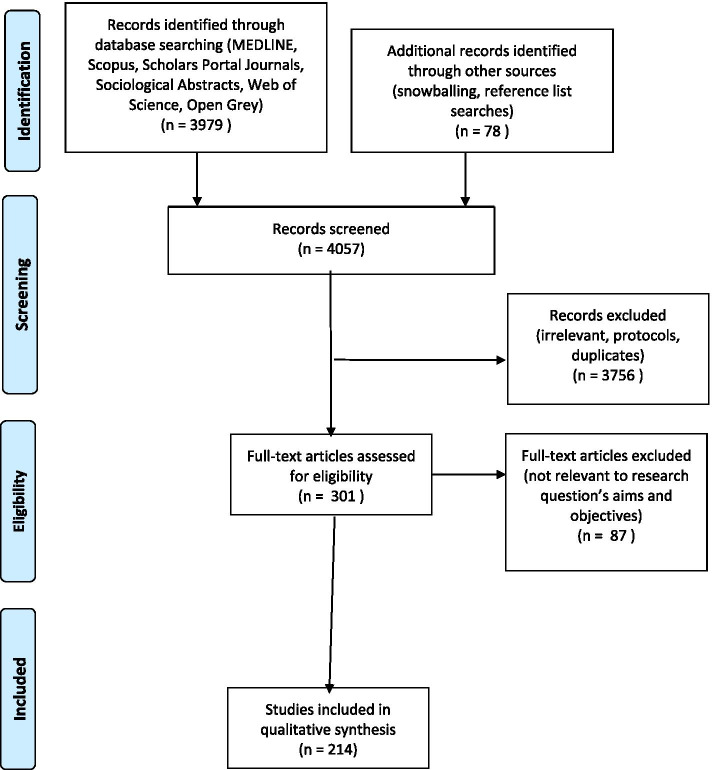
PRISMA Flow diagram

Key word searches were conducted on MEDLINE, Web of Science citation indexes for science and social science, Scopus, Scholars Portal Journals, Sociological Abstracts databases, as well as grey literature searches on Open Grey, and a search of citations in key studies. The University of California, San Francisco Social Interventions Research & Evaluation Network (SIREN) resources database was also searched. The search strategy is available in Appendix (Fig. 1).

After initial screening, full papers were reviewed and screened by one author (JP) against the inclusion criteria to determine eligibility. As this is a scoping study, not a systematic review, there was limited assessment of methodological quality. Initially all types of peer-reviewed papers were included, including qualitative, quantitative and mixed-methods studies of interventions, clinical decision-making tools, systematic and scoping reviews, and commentaries and opinion pieces. Study protocols were excluded. Any new and potentially relevant sources identified from citation lists were added. Upon review of full text, those that were then deemed outside the scope of the study were removed.

### Analysis

Each study abstract was scanned first to identify main themes. This was an iterative process which required going back and forth to capture multiple themes across different papers. Once these initial themes were identified, they were grouped into the following categories: literature reviews and systematic reviews; screening tools, economic needs-specific interventions; and service facilitators and barriers. Working through each category, the papers were analyzed to find points of commonality and divergence, and to identify any new emerging themes. We adopted qualitative content analysis to explore emerging themes, collapsing and expanding them over the course of the analysis process, until logical and clear themes and sub-themes emerged. This subjective interpretation of content using a key word coding system worked well to wrangle such a large body of literature into a workable volume of analysis to understand the phenomena under study [63, 64].

## Results

In total, the searches yielded 3979 results, and the titles and abstracts were initially screened for relevance. Duplicates were removed, and the remaining 214 were included in the review.

The following section will present the themes identified in the analysis. Unmet financial needs can be determined by the inability to access the necessities of life, in particular adequate, secure housing and stable supply of food. Financial need has been expanded to include other needs which are a direct result of inadequate finances, including stable housing and food security. It was common for papers to straddle two or more themes, for example, for one paper to include a screening tool description, service user perspectives, and to focus on food insecurity.

Analysis of this literature identified that although social needs interventions have existed for at least two decades in various forms, they have grown rapidly, as the vast bulk of the literature had been published in the past 5 years, indicating that social needs interventions are proliferating in line with a broader trend towards integrated behavioral healthcare, notably in the US and UK [35, 44, 65, 66]. Studies of interventions use a broad range of outcome measures, including those related to process, health impact, costs, and service user and provider perceptions. They also use a variety of terminology to describe such interventions, such as social prescribing [67], clinical-community linkages [68], and social referral [69]. The results are presented under three headings, each of which describes a different aspect of the process of financial needs intervention in primary care settings. Screening tools and screening tool evaluations covers the process of identifying patients with unmet financial need, and includes the tools themselves as well as analyses of their use and utility. Economic needs-specific interventions refers to interventions that occur in the primary setting, either to directly provide services or to refer patients to other service providers, and they are grouped under sub-categories for medical-legal partnerships; work, employment and welfare rights; food insecurity; and housing. Finally, the section on service users and service providers explores their respective perceptions of both facilitators and barriers to such interventions in the primary care setting.

### Screening tools and screening tool evaluations

Social and economic needs screening tools for use in primary care have proliferated in the past two decades [48]. Screening alone cannot address unmet social and economic needs, but it is key to understanding the patient in both social and medical dimensions [70]. Screening tools can range from one question [71, 72] to multi-dimensional, detailed questionnaires [73, 74].

Screening toolkits have been designed for multiple delivery modes. They can be completed by the patient by themselves or in concert with a clinical or non-clinical staff member before or during the encounter [75]. There are paper-based and digital formats for many screening tools (Table 1). There are proprietary tools designed by the primary care practices that use them, ready-made tools from national organizations, or externally sourced from other organizations such as community legal practices. With a large number of tools available, it can be feasible to adapt existing tools to local need, rather than reinventing the wheel and customization is the norm [26, 76, 77].

**Table 1:** Social and economic needs screening tools

**Name**	**Format**	**Source**	**Citation**
Centers for Medicare & Medicaid Services Accountable Health Communities Screening Tool	8-page questionnaire with sections on living situation, food, transport, utilities, safety, financial strain, employment family and community support, physical activity, substance use, mental health and disabilities	Centers for Medicare & Medicaid Services https://innovation.cms.gov/files/worksheets/ahcm-screeningtool.pdf	Billioux A et al., 2017 [78]
CLEAR	4-page, 4-step guide for front-line health workers Step 1: Treat Step 2 Ask (with suggested questions on e.g., employment, food and housing) Step 3: Refer (with suggested referral pathways) Step 4: Advocate (with suggestions for influencing community-level change)	CLEAR Collaboration, McGill University https://www.mcgill.ca/clear/files/clear/clear_toolkit_2015_-_english_1.pdf	Naz A et al., 2016 [79]
Health Begins Upstream Risk Screening Tool	4-page questionnaire with sections on education, employment, social connection and isolation, physical activity, immigration, overall financial strain, housing insecurity, food insecurity, diet, transportation, exposure to violence and stress with different questions for first visit and annual follow-up	https://www.aamc.org/system/files/c/2/442878-chahandout1.pdf	Bleacher H et al., 2019 [76]
Health Leads Social Needs Screening Toolkit	21-page guide to creating a screening toolkit with suggested domains and questions, tips and a sample one-page screening questionnaire in English and Spanish	Health Leads https://healthleadsusa.org/resources/the-health-leads-screening-toolkit/	Health Leads [73]
Medical-Legal Partnership Screening Guide	6-page screening guide template with sections in income, housing an and utilities, education and employment, legal status personal and family stability,	National Center for Medical-Legal Partnership https://medical-legalpartnership.org/screening-tool/	National Center for Medical-Legal Partnership [80]
Patient-Centered Assessment method	2-page questionnaire for health care provider to document assessment of patient’s health and well-being, social environment, health literacy, and required support	National Health Service https://njl-admin.nihr.ac.uk/document/download/2012029	Maxwell M et al., 2018 [81]
Poverty: A Clinical Tool for Primary Care Providers	2-page document with 3 steps: Step 1: Screen everyone with the question: do you have difficulty making ends meet at the end of the month? Step 2: Consider poverty as a disease risk factor Step 3: Intervene to ask every patient if they have filled out their tax forms (required for benefits access) and suggested questions for specific at-risk groups	Centre for Effective Practice https://portal.cfpc.ca/resourcesdocs/uploadedFiles/CPD/Poverty_flow-Tool-Final-2016v4-Ontario.pdf	Centre for Effective Practice, 2016 [82]
Protocol for Responding to an Assessing Patients’ Assets, Risks and Experiences (PRAPARE)	2-page questionnaire with sections on family and home, money and resources, and social and emotional health	National Association of Community Health Centers https://www.aapcho.org/projects/prapare/	National Association of Community Health Centers, 2016 [74]
Total Health Assessment Questionnaire for Medicare Members	4-page questionnaire with sections on physical and mental health, living situation an education	Kaiser Permanente https://sirenetwork.ucsf.edu/tools-resources/mmi/total-health-assessment-questionnaire-medicare-members	Kaiser Permanente, 2017 [83]
Well Child Evaluation Community Resources (WE CARE)	2-page, 10 question questionnaire on education employment, alcohol and drug use, safety, food and housing insecurity	Garg A et al., Johns Hopkins University School of Medicine, Baltimore, MD	Garg A, et al., 2007 [84]
WellRx Questionnaire	0.5 page questionnaire with 11 questions on unmet material needs, requested help for specific needs (e.g. finding employment, accessing education) and safety	Page-Reeves J et al., University of Albuquerque, NM	Pages-Reeves, J et al., 2016 [85]
Your Current Life Situation	2–5-page questionnaire with sections on current living situation (e.g., housing and food insecurity) and health behaviours (e.g., alcohol and drug use	Kaiser Permanente https://sirenetwork.ucsf.edu/tools-resources/mmi/kaiser-permanentes-your-current-life-situation-survey	Sundar KR, 2018 [86]

Among the evaluations and critiques of social needs screening tools, Gottlieb et al’s 2016 randomized controlled trial (RCT) was the first to show that in-person navigation for social needs is associated with families reporting decreased social needs, and significantly improved caregiver-reported child health [87]. However, the accuracy of screening tools to assess social needs is largely unevaluated [88], with WE CARE and Kaiser Permanente’s Your Current Life Situation notable exceptions [86, 89].

Complexity is not necessarily an advantage, particularly if the tool is designed for implementation by the health care provider during a patient encounter [78]. In a pilot study of a one-question poverty screening tool, the question:–“Do you ever have difficulty making ends meet at the end of the month”–had 98% specificity and 68% sensitivity in predicting a patient’s poverty [90]. Similarly, patients in a Virginia general internal medicine and emergency departments completed a 60-s survey to identify their unmet social needs, and the survey was effective in identifying the three most pressing unmet needs of the community the hospital serves [91].

In the studies focusing on food insecurity there was a definite tilt away from in-person screening, but for broader social needs screens, the findings from the literature were more mixed. How information is elicited can affect the screening outcome. On one hand, unstructured data collection can help reveal patients’ more complex needs. On the other, bias and stigmatizing selection of patients for screening may reduce the tool’s efficacy for detection of unmet social needs [92–94]. Other studies have demonstrated the acceptability of screening tools to patients [81, 95]. These are explored below under service user perceptions.

### Economic needs-specific interventions

#### Medical-legal partnerships

Medical-legal partnerships are a response to the clear association between health and socioeconomic risks that are amenable to legal interventions [96]. Through a collaborative intervention they typically embed civil legal aid professionals in the clinical setting [97]. Clients’ common presenting issues include problems with housing (including energy security), and income [96, 98, 99]. In the selected literature, almost all of the medical-legal partnerships are in the US. Outside the US, similar partnerships have been established in Canada [100, 101]. Given their mandate to provide primary care services in underserved areas, it is no surprise that CHCs are a natural home for health justice interventions and are where the number of medical-legal partnership services is growing the fastest [102–104]. Such services have a proven track record of helping clients obtain access to external food and income supports, claim unpaid welfare benefits, and prevent shut-offs of utilities [96, 99, 105].

#### Income, employment and welfare rights

Food insecurity, insecure and substandard housing and poverty-related legal issues are the expression of a more fundamental problem of income inadequacy. With that in mind, income is not as common a screening question as might be expected [106]. However, income issues are seen by welfare rights service providers as a good fit for their skill set and is often flagged as a presenting issue [96, 105, 107–109].

Studies of welfare rights services in primary care in the UK in the early 2000s reported increases in income for service users, as well as better self-reported mental and emotional health (although with only modest health improvements) [32, 110–116]. However, there is little evidence to date on the health impact of such interventions [117, 118]. At the forefront of this work in Canada is the St Michael’s Hospital Academic Family Health Team Social Determinants of Health Committee, which since 2013 has introduced numerous anti-poverty interventions, including socio-demographic screening and data collection, an income security health promotion service, a medical-legal partnership, a decent work initiative and a child literacy programme. Since 2013, the hospital’s Income Security Health Promotion service has assisted clients to improve their income and reduce expenses, but the papers reviewed did not reveal and evidence of measurable health impact [119–122]. Similar to their US counterparts, Canadian CHCs have a built-in mandate to address the upstream determinants of health [123]. A recent social prescribing pilot run across 11 CHCs in Ontario, which included financial needs interventions, to some extent formalized what CHCs are already doing in their respective communities [124–126].

In the UK, within its broad mandate of social prescribing (whereby the direction that the service takes is tailored to the needs identified by the client) the Bromley by Bow model includes welfare rights and employment support [127]. In 2016, the social prescribing service offered by Bromley by Bow was rolled out across the London Borough of Tower Hamlets via 37 general practitioner practices [128]. In Scotland, The Deep End Advice Worker Project–one of a collection of Deep End Project activities of General Practitioners at the Deep End, serving the 100 most deprived communities in Scotland–brings advice services to two of the most socially deprived areas of Glasgow [129, 130]. Noteworthy is that the model is one of assimilation, to embed the advice worker into the primary care practice, rather than co-locate services. Embedding the advice worker into the care team enables the service to increase its reach and benefit from the established relationship of trust between a patient and doctor, and between the advice workers and primary care physicians [131, 132]. As for employment, income interventions targeting employment are scarce and mainly focused on patients with mental illness [133].

#### Food insecurity

Food insecurity has been associated with adverse health outcomes [134, 135]. Leonard et al’s examination of overlapping clusters of food insecurity and poor health are suggestive of “shared causal mechanisms”, and the American Academy of Pediatrics (AAP) recommends screening for food insecurity [136–138]. Notably, food insecurity was one of the most common topics in the literature under review, with numerous dedicated screening tools, as well as frequent inclusion in wider screens, indicating that it may be seen as particularly amenable to intervention in the primary care setting [71, 137, 139–143]. It can present both challenges and opportunities for providers, including administration issues; a practice champion or advocate may be helpful in overcoming these challenges [144–146]. Although food insecurity screening may present an opportunity for further exploration of a patient’s social needs when asked in person, eliciting the information via a paper or digital questionnaire captures more revealing answers, reflecting the stigma associated with being unable to provide food for oneself or one’s family [137, 141, 145, 147–149].

A review of 29 peer-reviewed studies on food insecurity interventions either alone or in combination with other interventions identified three typical mechanisms: passive or active referrals to community and/or government agencies; vouchers for use at fresh produce outlets; and direct provision of food either by delivery or through an on-site food pantry [150]. It is, however, uncommon for studies to evaluate the outcome in terms of health or service utilization [151]. It is notable that food insecurity interventions are prevalent in the US and also in Canada. One explanation is that addressing food insecurity is in some ways a ‘quick win’: it is quick and easy to detect in screening and document, and can be directly addressed with referrals to food banks or even on-site food pantries. This is far more achievable in a primary care setting than tackling upstream causes, i.e., income insecurity, and is line with the proliferation of municipal-level food based interventions [152].

#### Housing

Lack of access to adequate housing is known to contribute to poor health [153, 154]. Housing, sometimes described as housing instability or homelessness, is frequently identified in the literature on screening and intervention for unmet social needs [155–160]. Housing security status is a common component of social needs screening tools [49, 69, 72, 89, 98, 161, 162]. Knowing that a patient is homeless or unstably housed can have an influence on clinical decision making [163–166]. Stabilizing housing is a key aim and outcome of inter-professional interventions and medical-legal partnership programmes [21, 96, 97, 107, 167–171].

An interesting aspect of health system interventions to address social needs is their involvement in creating affordable housing. Most interventions of this kind to date have been in the hospital setting rather than primary care, but they reflect a general growing awareness of the intertwined relationship between housing and health, and the merits of a Housing First approach [172–179]. It remains to be seen whether this interest in direct intervention in the form of affordable housing emerges in the primary care sector too.

### Service user and service provider perceptions of facilitators and barriers

#### Service users

While studies of primary care providers report fearing that they will create unrealistic expectations for their patients, other studies have found that on the contrary, patients understand the limitations of what their doctors can do to address their social needs, but nevertheless appreciate their efforts to do so, feel cared-for and find screening for social needs acceptable [39, 147, 154, 180–183]. However, this requires that broaching the subject of social needs be done with sensitivity to patients’ feelings of stigma, and fear of being reported to social service agencies if, for example they disclose that they do not have enough food to feed their children [148, 183]. Some studies report that patients welcome in-person help, whilst others prefer screening and referral modalities that are not face-to-face which can help overcome barriers of stigma [148, 160, 184, 185]. Patients do not always want their primary care providers to act on the identified unmet social needs [162, 182].

#### Service providers

Social needs screening is valued by physicians, as a way to improve their understanding of their patients [45, 147, 162, 181, 186]. In the US, for CHCs, screening often formalizes what they are already doing [187–189]. However, even among motivated physicians, uptake of screening can be low, unless it is routine and/or mandatory [154, 190–192]. Successful implementation relies on staff buy-in, training, integration into clinic workflows, and, for the best effect, a clinical champion [166, 193–196]. It requires the service provider to overcome ignorance about patients’ lived reality of poverty, push past discomfort with asking potentially stigmatizing questions, and having the communications skills set to do so [94, 197, 198].

Whereas primary care has strong linkages with other parts of the health system, linkages with social services are weak, navigation is complex and can hinder primary care providers’ efforts at referral [199]. Implementation of a social needs screening and assistance process can be challenging and resource-intensive [162, 197]. Facilitators include physical proximity, clear pathways for referral and a sense of mutual respect and shared aims, as well as practical considerations such as allocation of time away from clinical duties [196, 200].

A common theme in the literature is the key role played by a patient navigator. There are many terms to describe this connector role, including link worker, community-links practitioner, income security health promoter, family specialist, and care navigator [33, 53, 201–203]. The connector can also help bridge the gap between the norms and values of medical practitioners and the social services sector, improve physician satisfaction and help prevent burnout [154, 196, 204–210]. For the connector, common challenges include boundary setting and managing client expectations. Facilitators of success include lived experience of poverty, training and active buy-in from care providers [204, 211].

## Discussion

By far the most numerous were papers on interventions in the US. Apart from the sheer size of the population and complexity of the country’s health systems, several possible reasons for the preponderance of interventions emerged from the literature. Firstly, professional and government bodies including the American Academy of Pediatrics, and the American Academy of Family Physicians have participated in the call for physicians to address SDOH [212–214]. Both the National Association of Community Health Centers, and the Centers for Medicare and Medicaid Services have produced SDOH screening tools [49, 74, 78]. Secondly, there are readily identifiable suitable venues for SDOH interventions, including CHCs serving Medicaid recipients and the uninsured [170, 215–217]. Paediatric clinic settings, where children typically have regular check-ups together with a caregiver, have also been a key site for such interventions in the US [84, 89, 210, 218–220]. Thirdly, there are favourable funding mechanisms, financial imperatives and incentives. The metric of hospitalization cost savings–with broader positive similar findings– was used in several studies under review [167, 221–226]. Both Medicaid Managed Care Organizations (MMCOs) and Medicaid Accountable Care Organizations (ACOs) are actively involved in addressing SDOH [227–230].

Although social needs screening and interventions in primary care have really taken off in the past five to 10 years, they have a longer history than this trend would suggest. There were previous trends in this direction more than 20 years ago, such as the work done by family physicians in the UK to partner with welfare rights providers [108, 231]. Similarly in Canada, as early as 2001, the health sector was identified as a forum within which poverty could be addressed in Canada, and since then it has been the source of a series of interventions to address poverty among patients [19, 232].

Whatever the organizational structure, the ability to code and bill for non-medical services is key, and this is particularly apparent in the US [188]. In order to bill for services, health providers must typically input an International Statistical Classification of Diseases and Related Health Problems (ICD) code [233]. In the 10th ICD revision, there are 10 codes that relate to a patient’s socioeconomic and psychosocial circumstances . However, the codes are somewhat of a blunt instrument, and the existence of a billing code does not in itself guarantee that a service related to it will be billable [234].

For any intervention to gain traction, either with policymakers for funding, or with those involved to be onside, it is essential to be able to make a case for it, and to make a case that is stronger than that for competing priorities. Producing quantitative data to support advocacy for interventions such as the ones discussed in this paper is challenging, because it is difficult to determine valid metrics. Measuring the health impact of SDOH is it itself difficult, let alone interventions to address them. With so many comingled and intersecting factors, it is hard to tease out the effect of one thing or another. Moreover, health improvements may manifest over a long time, making them difficult to measure within the time constraints of a pilot project for example.

Measuring success has proven challenging, and the literature to date suggests a number of tensions with regard to evaluation. Is patient self-reported well-being a good enough metric to define a programme’s success? Are changes in health service utilization an adequate proxy for changes in health itself? Do health interventions have to yield benefits that are visible to the health sector to be deemed worthy of funding, or considered successful, or could the benefits accrue more tangentially, such as through decreased burden on the social welfare or justice system, or better educational outcomes? These are all issues that have yet to be explored in the literature.

While patient perspectives on income interventions have been examined, so far the emphasis has been on provider-led interventions. There is clearly more scope for more experimentation community-led interventions (such as those from the Bromley by Bow Centre in London), and more analysis of them.

## Strengths and limitations

Whilst other studies have examined some of the themes covered in this scoping review, this is the first to take such a broad sweep of the landscape of interventions targeted towards patients’ poverty, and to consider experiences across different countries, rather than focusing solely on the US. However, by only searching for publications in English, it may miss peer-reviewed studies and other grey literature published in other languages. The authors are aware, for example, of social needs screening tools that have been implemented in Japan, but this data could not be included under the inclusion criteria because it was only available in Japanese. As this is a scoping review, there is little examination of progamme efficacy and the sample for this descriptive review is non-random, comprised as it is of interventions that have attracted the interest of some academic researchers. This may create a biased view.

## Conclusion

There is a wide range of tools and processes in use to identify patients experiencing poverty, and address their economic needs, ranging from those tailor-made to an individual health practice, or to address one specific dimension of SDOH, to wide-ranging protocols that collect rich sociodemographic data. Primary care-based interventions that aim to address patients’ income needs operate at all levels, from passive sociodemographic data collection upon patient registration, through referral to external services, to direct intervention in addressing patients’ social insecurities such as providing on-site services including welfare system navigators and food pantries. The decision to undertake this work requires courage on the part of health care providers, because it can be difficult, time-consuming and complex. Success often relies on management buy-in and a practice champion. However, it is often appreciated, even when the scope of action available to health care providers is quite narrow. Economic needs interventions are typically found in settings with an identifiable patient population likely to have high unmet needs, with the number, scope and sophistication of programmes and interventions greatest in the US. Barriers to implementation include not just cost and time, but also navigating three different things: the complexity of social welfare system, the difficulty of billing for non-clinical services, and both patients’ and care providers’ emotions about what can be stigmatizing topics. Success is defined widely, from patient satisfaction, to health outcomes, but data on health outcomes is not widespread.

## Recommendations for future studies

There are several areas for potential future research. Firstly, the health impact of primary care-based economic interventions is a nascent field of investigation, and more research is needed to better investigate this. Secondly, the natural progression from individual-focused interventions, to those whereby the health care system engage at the community level to address upstream determinants such as lack of affordable housing and other infrastructural inadequacies, will be an interesting field of study [20]. Thirdly, the impact of COVID-19 on economic needs interventions in primary care, including the impact of remote service delivery modalities, is worthy of investigation.

## Data Availability

The datasets used and/or analysed during the current study are available from the corresponding author on reasonable request.

## Appendix

### Search strategy

#### The following the search strategy for the MEDLINE database

As per the iterative nature of the scoping review methodology, searches change in order to reflect emerging insights. Accordingly, the search strategy presented here represents the most fruitful searches.

A search for published literature was performed by one of the authors (JP) on August 7th and 8th 2020 on MEDLINE (via OVID) The search was limited to English language publications, and to studies on human and not animal subjects, published between January 12,000 and the date of the search (i.e., August 7th or 8th 2020).

Key word searches comprised a combination of social and health key words. MEDLINE searches were conducted iteratively, using Boolean operators, starting with ‘primary care’ AND ‘social determinants of health’. Subsequent searches took the first term and paired it instead with a number of terms identified through emerging analysis or from my previous research in this area: food insecurity, housing insecurity, income security, social needs, social needs screening, social assistance, social welfare, social prescribing, welfare rights, and link worker.

As the search progressed, the first search term was also expanded to ‘primary care OR family practice OR health cent*’ to capture literature that may only use these synonyms for primary care. Pairing ‘primary care OR family practice OR health cent*’ with ‘poverty’ and ‘screening’ or ‘intervention’ yielded hundreds of results (394 for screening 537 for intervention) but few (16 in total) studies that were relevant and had not already been captured.

As the iterative search process progressed, searches yielded many results, there were very few of relevance that had not already been captured, indicating that the searches initially conducted were successful in capturing most of the potentially relevant studies. The below Table 2 presents the most fruitful searches.

**Table 2:** MEDLINE search strategy and main results

**Search term**	**Search term**	**Search term**	**No of results**	**Excluded: not relevant/duplicates**	**Included for further screening**
**‘primary care’ AND**	**‘social determinants of health’**		**389**	**321**	**68**
	**‘social welfare’**		**119**	**112**	**17**
	**‘social needs’**		**160**	**136**	**24**
	**‘social prescribing’**		**44**	**13**	**31**
	**‘food insecurity’ AND**	**‘screening’**	**42**	**14**	**28**
	**‘social needs screening’**		**8**	**0**	**8**
	**‘welfare rights’**		**7**	**0**	**7**
	**‘housing insecurity’**		**6**	**6**	**0**
	**‘income security’**		**5**	**5**	**0**
	**‘social assistance’**		**17**	**17**	**0**
	**‘link worker’**		**12**	**12**	**0**
**‘primary care’ OR ‘family practice’ OR ‘health cent*’ AND**	**‘poverty’ AND**	**‘screening’**	**394**	**387**	**7**
	**‘poverty’ AND**	**‘intervention’**	**537**	**528**	**9**
	**‘social determinants of health’ AND**	**‘screening’**	**97**	**75**	**22**
	**‘social determinants of health’ AND**	**‘intervention’**	**86**	**86**	**0**
	**‘social needs’ AND**	**‘intervention’**	**38**	**36**	**2**
	**‘housing’ AND**	**‘intervention’**	**104**	**104**	**0**
	**‘food security’ OR ‘food insecurity’ AND**	**‘intervention’**	**387**	**387**	**0**
	**‘economic stability’**		**5**	**5**	**0**
	**‘employment’ AND**	**‘intervention’**	**212**	**212**	**0**
	**‘social needs’ AND**	**‘screening’**	**31**	**31**	**0**
**Total**			**2700**	**2487**	**223**
